# Amino Acid Profiling and Chemometric Relations of Black Dwarf Honey and Bee Pollen

**DOI:** 10.3389/fnut.2020.558579

**Published:** 2020-12-07

**Authors:** Sarana Rose Sommano, Farhan M. Bhat, Malaiporn Wongkeaw, Trid Sriwichai, Piyachat Sunanta, Bajaree Chuttong, Michael Burgett

**Affiliations:** ^1^Plant Bioactive Compound Laboratory, Department of Plant and Soil Sciences, Faculty of Agriculture, Chiang Mai University, Chiang Mai, Thailand; ^2^Cluster of Agro Bio-Circular-Green Industry (Agro BCG), Chiang Mai University, Chiang Mai, Thailand; ^3^Programme of Food Production and Innovation, Faculty of Integrated of Science and Technology, Rajamangala University of Technology Lanna, Chiang Mai, Thailand; ^4^Meliponini and Apini Research Laboratory, Department of Entomology and Plant Pathology, Faculty of Agriculture, Chiang Mai University, Chiang Mai, Thailand; ^5^Department of Horticulture, Oregon State University, Corvallis, OR, United States

**Keywords:** Asian honeybee, flower by-product, microscopy, protein, pollen grain

## Abstract

This research reports the characterization of bee pollen of *Apis andreniformis* colonies on the basis of morphology, proximate composition, the amino acid, and nutritive patterns in relation with their honey. The pollen gains of the sampling colonies revealed variations in their structure, symmetry, and sculpture. The exile surfaces of the pollens showed psilate, scabrate, clavate, and echinate types of morphology. Total amino acid content of black dwarf honeybee collected pollen (150 mg/g) was found significantly higher than that of honey (15 mg/g) from the same colony. Threonine, phenylalanine, and leucine were among the highest essential amino acid types found in the analyzed pollen and honey samples. The proline content in both products was found the lowest comparing to other amino acid types. The moisture content of the honey samples were found to exceed the limit as prescribed by Codex Alimentarius Commission (<20%). The ash content of the analyzed samples was mostly within the limits (<0.6%) prescribed by international norms. The fat content of the pollens varied from 5.01 to 5.05%, and the honey showed zero fat content. The carbohydrate content in the honey samples was found to differ significantly from each other with a maximum content (73.16%), and the lowest carbohydrate content was 67.80%. The pollen and honey samples were found to have positive effect on *in vitro* digestibility of proteins.

**Graphical Abstract:**
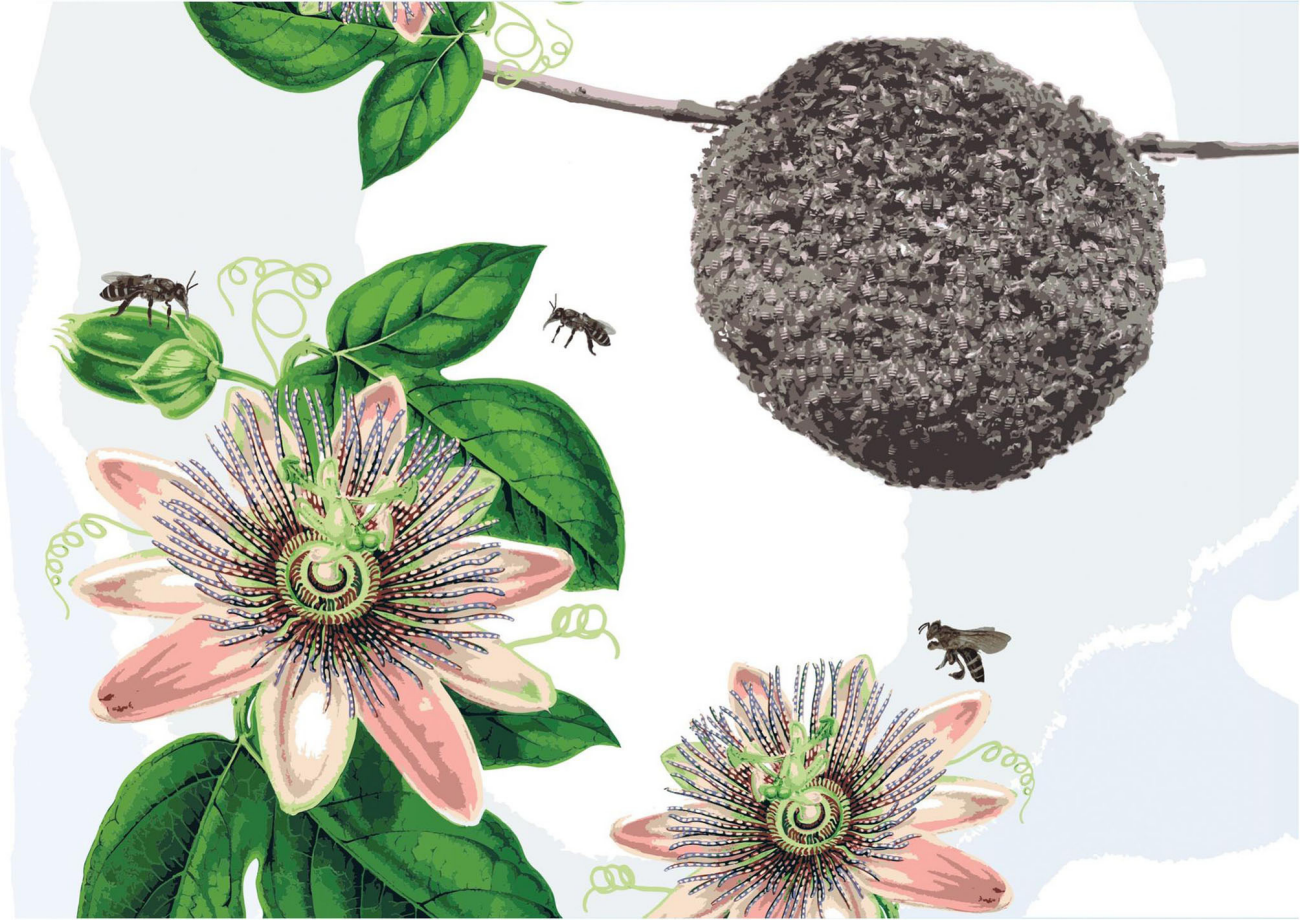


## Introduction

Floral pollen is the the product of flowers which is in essence packaged gametes for gymnosperms and angiosperms in the flowering plants (1). As part of plant pollination process, insects such as honeybees, collect the pollen which is essential in their diet (as main sources of proteins, vitamins, minerals, saturated fatty acids and oils, carbohydrates, and other healthy compounds such as antioxidants) (2, 3). After agglomeration process to form small spheres or pellets of the pollen with the combination of nectar and salivary secretion, the honeybee carries the pollen in a pellet combined with nectar and salivary secretions in the corbicula or pollen basket located on each hind leg, which is known generally as “bee pollen.” These pollen loads can be collected by the beekeeper at the hive entrance and are considered as a nutrient-rich healthy bee product apart from honey, royal jelly, and propolis for human consumption (4–6). Bee pollen is used in the apitherapeutic treatments as it demonstrates medicinal properties including antifungal, antimicrobial, antiviral, anti-inflammatory, and immunostimulating. It is also used as local analgesic and also facilitates post-operative wound and burn healing processes (2, 4, 7). Amino acids represent as little as 1% (wt/wt) of honey ingredients, and the main source of protein (up to 50%) and amino acids in honey is bee pollen (8). Pollen and amino acid compositions are specific for individual bee colonies at the same geographic locations, types of flowers, and environmental conditions; thereby, they are also used to discriminate the botanical origins of honey (8–12).

The use of bee pollen is common in the human diet and has been officially recognized as a medicine by the German Federal Board of Health. It is also known generally as the “only perfectly complete food” (2, 13, 14). The recommended daily intake of bee pollen as a dietary supplement is 5–10 g/d (3). The Food and Agriculture Organization (FAO) recommends bee pollen consists of more than 16 essential and non-essential amino acids including, for example, methionine (Met), lysine (Lys), threonine (Thr), histidine (His), leucine (Leu), isoleucine (Ile), valine (Val), and phenylalanine (Phe), whereas arginine (Arg), His, Ile, Leu, Lys, Met, Phe, Thr, and tryptophan (Trp) (7, 12, 15–17). Such ingredients have begun to be considered as functional products in food and feed supplements (18).

*Apis andreniformis* (Hymenoptera: Apidae) is one of the nine honey species distributed throughout tropical and subtropical Asia (19, 20). In Thailand, the species has been discovered from the coastal flats (1–100 m above sea level) of the east (Chanthaburi province) to high mountainous and forest areas at about 1,600-m altitude in the northern parts of Thailand (21). Because pollen is considered to be the main source of amino acids in honey, analysis of its content together with chemometric techniques is often used to estimate botanical and geographical origin of honeys (10, 22). To this end, *A. andreniformis* is patchily distributed, thus, very few studies regarding chemical compositions of its honey and pollen, providing the nutritional information, have been reported (23). The objective of this study is so to determine protein, amino acid compositions, and nutritional values of dwarf honeybee pollen in relation with those in the honey of the same sources. By providing chemometric relations of amino acid profiling, this study could be also used to describe the geographical identity of this exotic honeybee. To the best of our knowledge, this is the first report from black dwarf honeybee on the nutritional requirements of human and honeybees.

## Materials and Methods

### Sample Collection

Honey and bee-pollen samples were collected from five colonies of *A. andreniformis* of the same population size. Samples were collected during February–March 2019 within 5 km^2^ in an open highland forest (18°53.086′ N 98°50.8208′ E), Mae Rim district, Chiang Mai. The area was selected as the high altitude (800 m above sea level) that favors *A. andreniformis* and is distanced from industrial agriculture. Colonies were brought back to Meliponini and Apini Research Laboratory and the pollen was collected from pollen comb immediately, and they were then kept at −21°C until analysis (24). Honey was also harvested from the same colonies. All honey samples were extracted, unpasteurized, and stored at 4°C prior to the analysis (25).

### Pollen Morphology

Bee-pollen samples were morphologically identified under Primo Star microscopes (Carl Zeiss, Germany). A drop of 20% fructose solution containing 0.5 g crystallized phenol was added on top of the sample onto glass slide to facilitate the transferring and spreading out of pollens and to accelerate their swelling (25). The slides were then dehydrated on a hot plate at 40°C and mounted with glycerine gelatin before viewing under magnification.

### Amino Acid Analysis

Amino acid compositions were determined by an ARACUS amino acid analyzer (membraPure GmbH, Bodenheim, Germany) equipped with C18 column and refractive index (RI) detector. The high-performance liquid chromatography was coupled to a mass spectrometer equipped with electrospray ionization for amino acid separation followed by ninhydrin reaction and photometric detection. The detector was set at 570 and 440 nm, and N_2_ was used as inert gas. Acid hydrolysate extraction was performed according to the adapted method of Vanderplanck et al. (26), as well as for bound amino acids, 10 mL of extracting solution comprising 6 N HCl and 0.1% phenol was added to 30 mg pollen (dried weight) or 300 mg honey. The hydrolysate was accomplished under TANK PRO microwave digestion unit (Sineo, China) for 2 h and left to cool at room temperature, under a fume hood. The sample was filtered through Whatman no. 1 filter paper. The clear hydrolysate (1 mL) was aliquoted and evaporated to dryness under rotary vacuum. Afterward, 1 mL of the sample buffer was added to dissolve the dried sample. Sample was filtered through 0.45-μm microfilter, and clean extract was injected (20 μL) into the amino acid analyser with the condition set up according to the manufacturer's instructions.

### Protein Quality Evaluation

#### Protein Content

Crude protein (% N × 6.25) was determined by the Kjeldahl's method using the nitrogen combustion instrument (FP828, LECO, USA) (27, 28).

#### Essential Amino Acid Index

The Essential Amino Acid Index (EAAI) was calculated using the method of Oser et al. (29), by using the ratio of relative essential amino acids in the test protein as compared to the respective values in whole egg protein (30).

(1) $$\begin{array}{l} EAAI=\;n\sqrt{\frac{Lys_{a}\times Tyr_{a}\times \ldots ..\times His_{a}}{Lys_{b}\times Tyr_{b}\times \ldots .\times His_{b}}} \end{array}$$

where a is the amino acid in test sample, and b is the amino acid in reference protein sample; *n* is the number of essential amino acids.

#### Biological Value

Biological value (BV) was calculated according to the equation used by Oser et al. (29).

(2) $$\begin{array}{l} BV=1.09\;\times \;Essential\;amino\;acid\;index-11.7 \end{array}$$

#### Protein Efficiency Ratio

As per the joint report of World Health Organization (WHO)/FAO expert's consultation, it was reviewed and suggested to replace the method of protein efficiency ratio assay using rat growth and had been concluded unsatisfactory. Thus, protein efficiency ratios (PERs) on the basis of interaction between Leu–proline (Pro) and Leu–tyrosine (Tyr) were calculated using the modified regression equations as described by Alsymer et al. (31).

(3) $$\begin{array}{l} PER-1=-0.684+\;0.456\left(Leu\right)-0.047\left(Pro\right) \end{array}$$

(4) $$\begin{array}{l} PER-2=-0.468+\;0.454\left(Leu\right)-0.105\left(Tyr\right) \end{array}$$

#### Amino Acid Score

Amino acid scores (%) for infants (preschool) and adults were calculated as the ratio of observed value of amino acid (g/100 g of protein) to the reference pattern as provided by FAO/WHO (32).

#### *In vitro* Digestibility Test

Dried sample (1.5 g) was mixed with 0.002% pepsin in 0.0075 N HCl solution (150 mL). The samples were incubated with continuous swirling for 16 h at 45°C. After filtration through Whatman no. 5, the clear solution was analyzed for protein content (1). Digestibility was calculated as the ratio of protein content of the supernatant to the original protein content of the sample (1.5 g). Results are expressed as digested protein content (g) /100 g bee bread (pollen) total protein (18).

### Proximate Analyses

Proximate analyses were performed according to the Association of Official Analytical Chemists methods (33). Total carbohydrate contents were calculated using the following equation:

(5) $$\begin{array}{l} \text{Carbohydrate content }\left(\%\right)=100-(\%\text{ moisture content}\\ \;+\%\text{ total protein}+\%\text{ ash content}\\ \;+\%\text{ totalfatcontent}) \end{array}$$

The total energy of one serving of sample (100 g fresh weight) was calculated according to the following equation (34):

(6) $$\begin{array}{l} \text{Total energy}=(\text{energy content of }1\text{ g protein}\\ \;\times \text{ g protein of sample})+(\text{energy content of }1\text{ g fat}\\ \;\times \text{ g fat of sample})+(\text{energy content of }1\text{ g carbohydrate}\\ \;\times \text{ g carbohydrate of sample}) \end{array}$$

Where the energy content of 1 g protein = 4 kcal, energy content of 1 g fat = 9 kcal and the energy content of 1 g carbohydrate = 4 kcal.

### Statistical Analysis

Data were collected from three independent replications and analyzed using the analysis of variance (ANOVA) with SPSS (version 23). The principal component analysis (PCA) was used to explain and interpret interdependence of data (1, 35). PCA and cluster analysis of all sample types and contents of amino acids were performed using XLSTAT (trial version, XLSTAT.com).

## Results and Discussion

### Pollen Morphology

**Figure 1:**
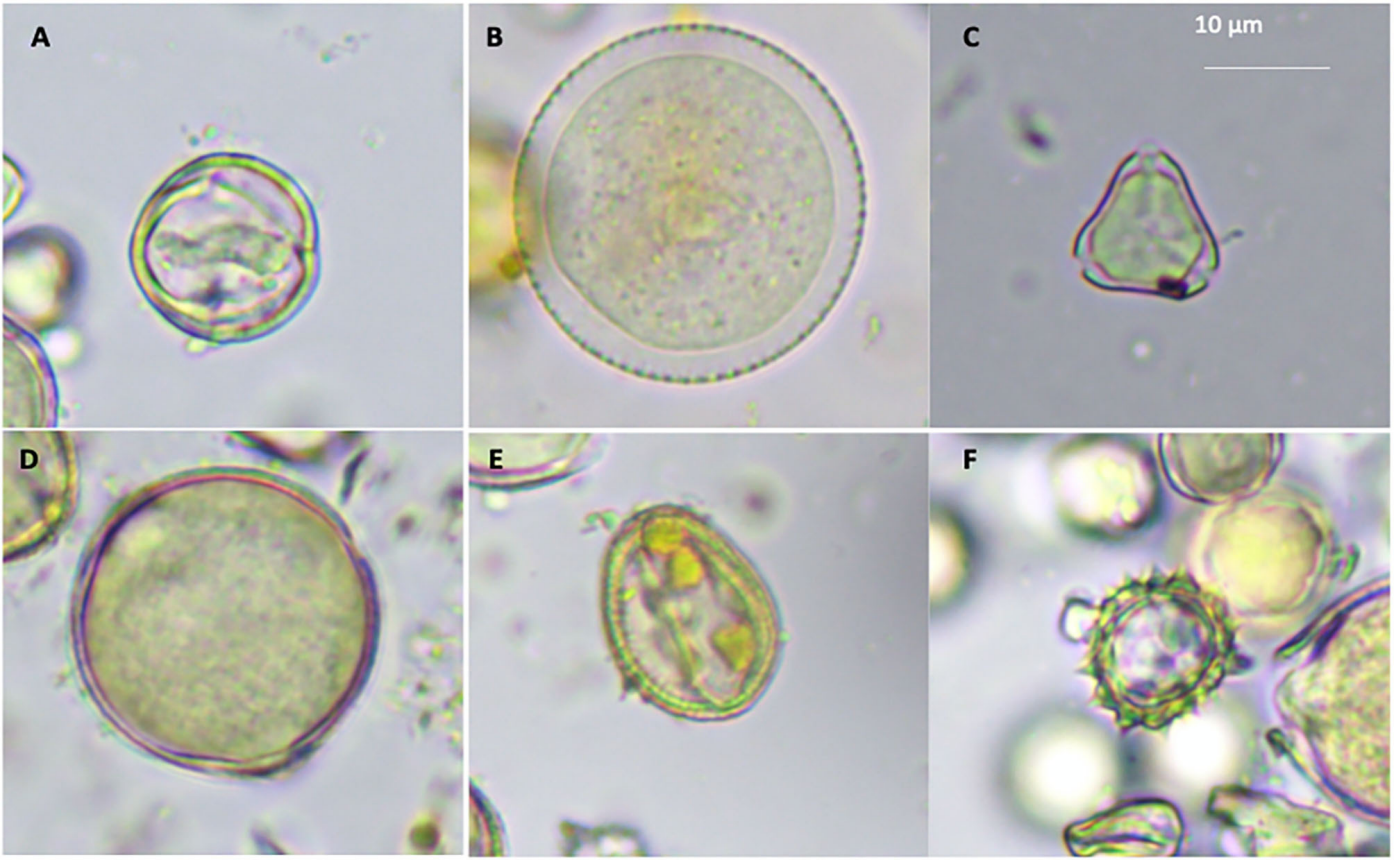
Pollen grains morphologies of *A. andreniformis*; monolete **(A)**, inaperturate **(B)**, trizonocolpate **(C)**, dicolpate **(D)**, monolate **(E)**, and trilete **(F)**.

Even though taxonomical description of pollen data base in South East Asia is not well-documented, three pollen batches (viz., those belonging to monofloral, bifoloral, and heterofloral) were typically defined (3). Several structures of pollen gains were identified from the sampled colonies, namely, monolete (Figure 1A), inaperturate (Figure 1B), trizonocolpate (Figure 1C), dicolpate (Figure 1D), monolate (Figure 1E), and trilete (Figure 1F). The exile surfaces were psilate (Figures 1A,C,D), scabrate (Figure 1B), clavate (Figure 1E), and echinate (Figure 1F) It is apparent that those of monolete are possibly from the Fabaceae such as mimosa or the Anacardiaceae family such as *Tapirira* species (3), whereas trizonocolpate was similar to those belonging to longan flowers (11, 36). Variation of pollen types was according to bee species, which largely associates with body size and flying distance. The bigger bee is able to visit more plant species and carry more pollen load (37). In the case of *A. andreniformis*, the variable of food resources seems to be less than other *Apis* spp. as they could forage later but in low numbers, presumably exploiting the resource behind productive time (38). The pollen morphology also indicates that types of pollen found in colony 3 are less variable than the rest of the colonies (Table 1). This then confirms the limitation of pollen sources for this honeybee species. Pollen and nectar are the two main sources of food such as sugars, protein, minerals, vitamins, fatty acids, and a small percentage of other components for honeybee (39). The morphology of pollen aids in the identification of the bee forage that could be useful in increasing the efficiency and commerciality of beekeeping industry. Hive stored pollen inherits mutualistic microbe or small community of microbes in a very acidic condition that causes pollen structure decompartmentation due to biochemical breakdown of the pollen resulting in easy releasing of nutrients for the bees. Deformation structure was also observed (Figures 1D–F), indicating degradation of cell wall, which largely contributes to the significance in protein content of pollen after digesting (1).

**Table 1:** The frequency of pollen types identified from colonies of *A. andreniformis*.

**Pollen types**	**Colony 1**	**Colony 2**	**Colony 3**	**Colony 4**	**Colony 5**
A (monolete-psilate)	+ + +	++	–	+	+
B (inaperturate)	+	+	–	+	+
C (trizonocolpate)	+	+	+	++	++
D (dicolpate)	++	+ + +	+ + ++	++	+
E (monolete- scabrate)	++	++	+	+	+
F (trilete)	+	+	–	+	–

+ + ++*Most frequent*, –*not found*.

### Amino Acid Analysis

The amino acid chromatograms of the bee pollen from different colonies and their respective honey are provided as supplementary. As shown in Table 2, total amino acid content of black dwarf honeybee pollen (~150 mg/g) was higher than that of honey (~15 mg/g) from the same colony. Thr, Phe, and Leu were among the highest essential amino acid types found. Surprisingly, the amount of these types of the amino acids was similar in both pollen and honey, with the Thr being slightly higher (~3 mg/g) than the others. The pollens, however, gave greater non-essential amino acid contents than those of the honey with glycine (Gly), γ-aminobutyric acid (GABA), and aspartic acid (Asp) were among the highest (~50 mg/g). Pro is usually the major amino acids of pollen grains and honey (8, 25, 41). Our finding however, suggest for this multiflora grains type collected by black dwarf honeybee and honey had far less Pro than other amino acid types. In other studies, the lower Pro content was claimed to represent that bee workers collected pollen and nectar at the beginning of the flowering seasons (8, 42). This also indicates the immaturity of the honey, particularly for those of *Apis mellifera* and *Apis cerana* (42, 43). Pro is a dominant amino acid that is considered an important indicator for determination of honey quality. Pro content in honey has been found to decrease constantly during storage, and its concentration might be used as an indicator of the honey ripeness as reported earlier by Berger et al. (44). All the analyzed honey and pollen samples except honey of CL2 showed values higher than the internationally accepted minimum value of 180 mg/kg for Pro (45, 46). Thus, the honey of CL2 could be regarded as immature honey. Honeybees are exclusive users of carbohydrates as metabolic fuel for high intensity of hovering flight. They also have the ability to oxidize the amino acid Pro, but the contribution of this amino acid is negligible as compared with carbohydrates. The enzymatic activity involved in Pro oxidation is lowest in honeybees and thus cannot oxidize it at a high rate (46).

**Table 2:** Amino acid compositions *A. andreniformis* bee pollen of different colonies from the natural habitat in the agroforest of Northern Thailand.

		**Colony 1**		**Colony 2**		**Colony 3**		**Colony 4**		**Colony 5**		**A**
		**Pollen**	**Honey**	**Pollen**	**Honey**	**Pollen**	**Honey**	**Pollen**	**Honey**	**Pollen**	**Honey**	
**Essential amino acids**												
Histidine^(1.5g/100g protein)^	His	—	—	—	—	—	0.56, 0.56	—	—	—	—	1.50
Isoleucine^(3.0g/100g protein)^	Ile	0.68, 0.09	—	0.453, 0.45	—	—	—	0.34, 0.34	—	—	0.015, 0.01	4.00
Leucine^(5.9g/100g protein)^	Leu	0.93, 0.22	0.244, 0.03	0.744, 0.03	0.94, 0.72	1.02, 1.02	7.89, 6.96	0.46, 0.46	0.34, 0.02	0.38, 0.38	0.17, 0.01	4.50
Lysine^(4.5g/100g protein)^	Lys	—	—	—	—	—	9.71, 9.70	—	—	—	—	3.00
Methionine	Met	0.28, 0.16	0.052, 0.01	0.457, 0.28	0.12, 0.10	1.58, 0.28	1.84, 1.67	0.53, 0.53	0.03, 0	0.74, 0.02	0.012, 0.01	1.50
Phenylalanine^(3.8g/100g protein)^	Phe	0.96, 0.01	0.502, 0.07	1.491, 0.11	1.41, 1.09	2.17, 1.02	1.34, 0.80	1.46, 0.15	0.55, 0.04	0.67, 0	0.27, 0.02	1.50
Threonine^(2.3g/100gprotein)^	Thr	1.18, 0.17	0.472, 0.06	1.42, 0.04	2.30, 1.8	4.41, 1.27	7.19, 4.64	1.7, 0.17	0.86, 0.05	1.28, 0.24	0.38, 0.03	1.50
Tryptophan	Trp	—	0.006, *0.01*	—	—	—	5.70, *5.70*	—	—	—	—	1.00
Valine ^(3.9g/100gprotein)^	Val	—	—	—	—	0.05, 0.05	2.60, *2.57*	—	—	—	—	4.00
**Non-essential amino acids**												
Alanine	Ala	0.73, *0.23*	0.008, 0	0.54, *0.07*	—	0.90, 0.23	0.35, 0.23	0.69, 0.09	0.002, 0	0.17, 0.17	0.003, 0	
Arginine	Arg	—	—	—	—	—	0.048, 0.05	0.091, 0.09	—	0.132, 0.13	—	
Aspartic acid	Asp	4.67, 1.18	0.230, 0.03	2.74, 0.07	0.62, 0.48	3.21, 0.29	0.38, 0.19	2.54, 0.12	0.16, 0.01	3.56, 0.20	0.105, 0.01	
Asparagine	Asn	26.9, 8.30	0.51, 0.06	29.12, 7.11	2.71, 2.09	41.95, 2.87	2.47, 0.77	34.23, 3.65	0.74, 0.05	29.29, 6.02	0.40, 0.03	
Cysteine	Cys	2.50, 1.10	2.28, 0.31	2.06, 0.09	7.79, 6.02	9.59, 5.67	7.08, 3.41	3.67, 0.69	3.60, 0.23	2.44, 0.09	1.51, 0.12	
Glycine	Gly	90.5, 22.7	5.21, 0.59	80.2, 17.3	24.01, 18.51	46.39, 7.75	10.01, 1.97	45.32, 2.64	5.74, 0.46	35.90, 3.49	4.84, 0.38	
Proline	Pro	2.96, 0.27	0.28, 0.03	3.68, 0.36	1.32, 1.02	4.62, 0.95	0.49, 0.49	2.51, 0.64	0.40, 0.04	3.30, 0.25	0.23, 0.03	
Serine	Ser	4.34, 0.73	0.38, 0.04	3.72, 0.09	1.98, 1.54	5.99, 0.66	26.78, 23.95	3.17, 0.30	0.70, 0.04	3.66, 0.20	0.35, 0.02	
Tyrosine	Tyr	—	—	—	—	—	0.19, 0.19	—	—	—	—	
Glutamic acid	Glu	9.44, 0.15	0.51, 0.06	4.54, 1.16	1.86, 1.44	3.15, 1.33	0.70, 0.70	5.86, 3.17	0.54, 0.04	9.54, 0.73	0.35, 0.03	
γ-Aminobutyric acid	GABA	38.7, 3.77	1.56, 0.22	42.57, 5.58	5.93, 4.53	81.0, 4.28	1.21, 1.21	45.57, 5.05	1.37, 0.07	33.84, 2.13	0.93, 0.04	
Taurine	Tau		0.096, 0.01	0.26, 0.02	0.60, 0.46	1.454	0.58, 0.47	0.14, 0.14	0.091, 0.02	0.23, 0	0.10, 0.01	
Total		184.77	12.24	173.99	51.59	206.03	87.12	148.28	15.12	125.13	9.67	
Total essential amino acids (%)		2.18	10.42	2.62	9.25	4.48	42.28	3.03	11.77	2.45	8.76	
Total non-essential amino acids (%)		97.82	89.58	97.38	90.75	95.52	57.72	96.97	88.23	97.55	91.24	
Total acidic amino acids (%)		7.64	6.04	4.18	4.81	3.08	1.24	5.66	4.63	10.46	4.70	
Total basic amino acids (%)		—	—	—	—	—	2.80	0.06	—	0.105	—	
Total aromatic amino acids (%)		0.519	4.15	0.857	2.73	1.05	8.299	0.985	3.637	0.535	2.792	
Leucine/isoleucine ratio (BCAA)		1.37	0.244	1.64	0.94	1.02	7.89	1.35	0.34	0.38	11.33	
EAAI		20.75	4.295	22.599	22.920	27.989	61.44	20.859	7.767	20.652	2.270	
BV (%)		10.91	−7.02	12.93	13.28	18.81	55.27	11.04	−3.23	10.81	−9.22	
PER-1		−0.399	−0.58	−0.52	−0.32	−0.44	2.89	−0.59	−0.55	−0.66	−0.62	
PER-2		−0.046	−0.357	−0.130	−0.04	−0.049	3.094	−0.259	−0.313	−0.295	−0.391	

*Values are mean of the triplicates + standard error. A = Minimal levels of essential amino acids required by honeybees (40)*.

From the nutrition standpoint, Thr is known to influence brain development of infacts and considered a second-rate essential amino acid in the maintenance requirement of human body (18, 47). Similarly with Thr, the elevated concentrations of Phe that occurs in mammal protein diet exceptional of human breast milk could cause brain disorder in early life of human (48). Leu plays the important task in muscle development and accelerating growth hormone (49). Gly and GABA are major neurotransmitters that help in the development of human central nerve system in adult mammal (50). Gly acts not only as a powerful inhibitory neurotransmitter but also paradoxically as a coagonist or modulator of the excitatory neurotransmitter glutamate at its receptors (51). GABA functions as inhibitory synapses in the brain by binding to specific receptors in the plasma membrane of both causing hyperpolarization there by targeted as anticonvulsant, anxiolytic, and sedative–hypnotic agents (52, 53). Besides the enrichment amino acids, bee pollen shows high content of sugars, unsaturated and saturated fatty acids, and minerals, which could have potential sources for dietary supplement (3). Other than these, bee pollen also possesses bioactivities including antimicrobial, antimutagenic, antioxidant, and anti-inflammatory. These bioactivities of pollen grains depend on the presence of active phytoconstituents (13, 54).

We also used two-way ANOVA to analyze significant variations of amino acid within these sources and within the colony samples as shown in Table 3. The data also showed that IIe was the only essential amino acid that was significantly different between the type of the sources, whereas the amounts of Ala, Asp, asparagine (Asn), Gly, Pro, Glu, and GABA non-essential amino acids were significantly different between honey and pollen. The essential amino acids were not significantly different between the collected colonies. Nonetheless, Ala, GABA, and Tau of non-essential amino acid types were detected in different amount from these colonies. Interaction between the sources and colony indicated that GABA was the key amino acid distinguishing pollen and honey for this particular type of wild honey. To further explain chemotypic relations in these samples, PCA was conducted from these datasets.

**Table 3:** Significance levels in two-way ANOVA of amino acid sources (pollen and honey) as affected by colonies of *A. andreniformis*.

	**Essential amino acid**										
	**Arg**	**His**	**Ile**	**Leu**	**Lys**	**Met**	**Phe**	**Thr**	**Trp**	**Val**	
Amino acid sources (S)	NS	NS	**	NS	NS	NS	NS	NS	NS	NS	
Colony (C)	NS	NS	NS	NS	NS	NS	NS	NS	NS	NS	
S × C	NS	NS	NS	NS	NS	NS	NS	NS	NS	NS	
	**Non-essential amino acid**										
	**Ala**	**Asp**	**Asn**	**Cys**	**Gly**	**Pro**	**Ser**	**Tyr**	**Glu**	**GABA**	**Tau**
Amino acid sources (S)	**	**	**	NS	**	**	NS	NS	**	**	NS
Colony (C)	**	NS	NS	NS	NS	NS	NS	NS	NS	**	**
S × C	NS	NS	NS	NS	NS	NS	NS	NS	NS	**	NS

NS, ^**^not significant (p < 0.05).

### Chemometric Analysis

**Figure 2:**
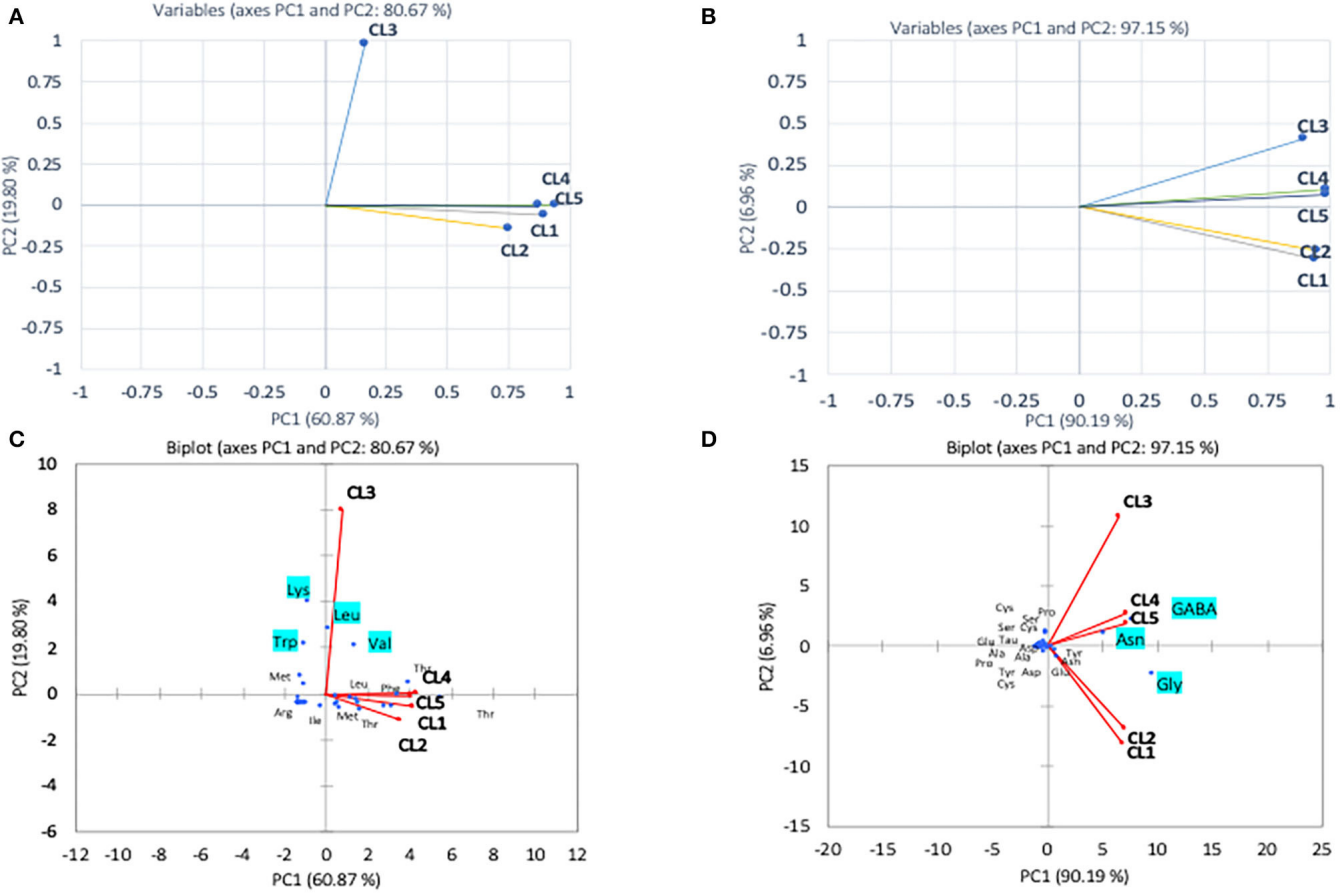
Principal component analyses of essential amino acid **(A)** and non-essential amino acid **(B)** compositions of different pollen from *A. andreniformis* colonies (CL1–CL5) data was also performed using biplot cluster analysis with types of essential amino acids **(C)** and non-essential amino acids **(D)**.

The multivariate (PCA) analysis was used on the amino acids determined in the research to differentiate pollen and honey on the basis of their botanical origin. The first (PC1) and second (PC2) principal components were found to depict 60.87 and 19.80% of variance in case of essential amino acids (Figure 2). The first component showing 60.80% of variance was mostly dominated by Met, Thr, Leu, and Phe, whereas the second component with variance 19.80% was dominated by Lys, Trp, and Arg. The PCA revealed that the first four loading factors accounted for 96.43% of the variance in essential amino acids, with first, second, third, and fourth components contributing 60.87, 19.80, 10.69, and 5.58% of the variance, respectively. The first component showing 60.87% variability was found to correlate positively with Ile, Leu, Phe, Thr, and Met, while as the second component accounted for 19.80 of variance exhibiting positive correlation with Leu, Trp and Val. The CL1 was found to correlate positively with CL4 exhibiting Pearson correlation coefficient of 0.824 and with CL5 having coefficient of 0.724. This was attributed to the closeness of cluster formed by CL1, CL4, and CL5. Among the colonies analyzed by PCA, CL3 was found to depict the least correlation coefficients of 0.07, 0.03, 0.14, and 0.12 with CL1, CL2, CL4, and CL5. This accounted for the separation of this colony from the rest of the colonies. The analysis non-essential amino acids by PCA revealed 99.84% of cumulative variance for the first four loading factors. The first component with 90.19% of variance was found to exhibit positive correlation with Asn, Gly, Glu, and GABA, whereas the second component (PC2) was found to show positive correlation with cysteine (Cys), Pro, serine (Ser), and GABA. The Pearson correlation revealed that CL2 correlates higher with CL1 exhibiting correlation coefficient of 0.95, and CL4 correlates significantly greater with CL5 (0.99) that enabled their closeness with respect to each other in the PCA plot.

**Figure 3:**
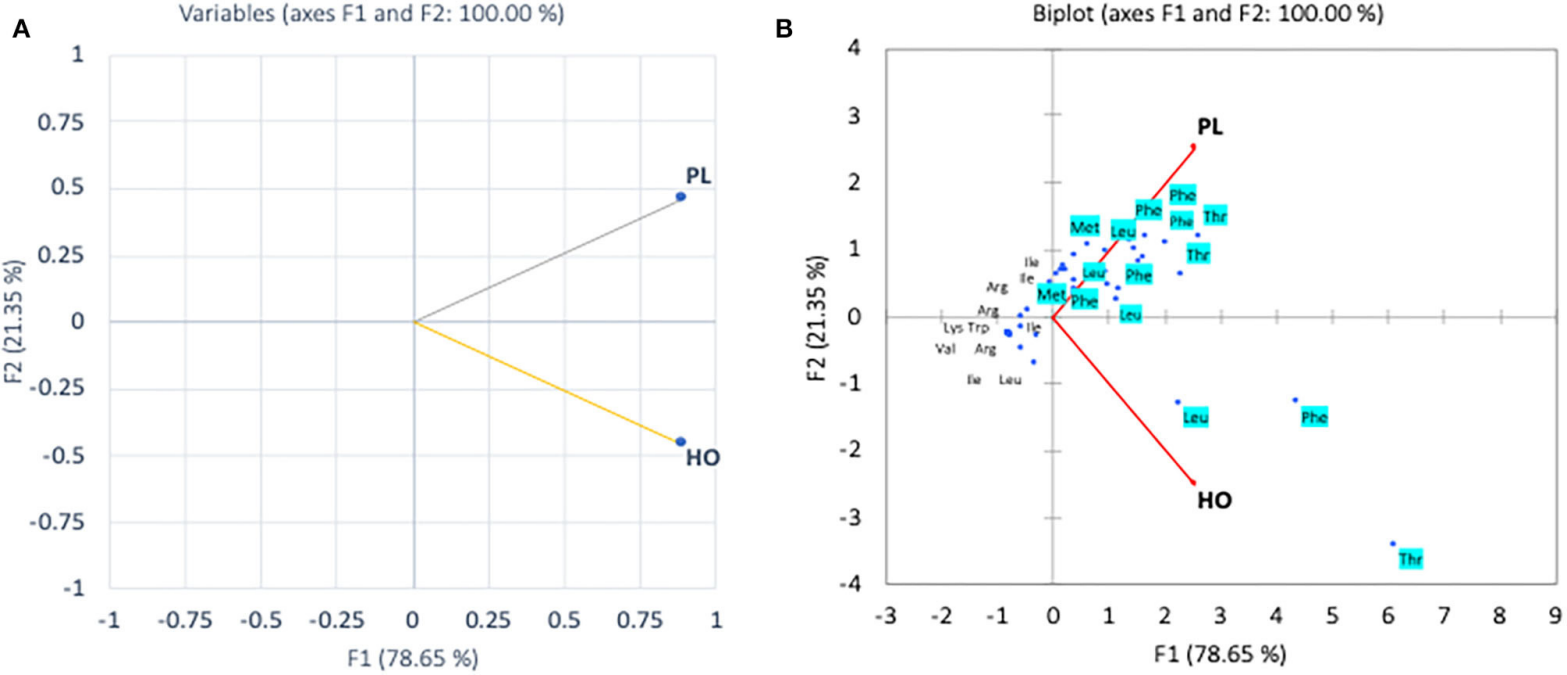
Principal component analyses of essential amino acid compositions **(A)** and biplot cluster analysis with types of essential amino acids **(B)** of pollen (PL) and honey (HO) of *A. andreniformis*.

**Figure 4:**
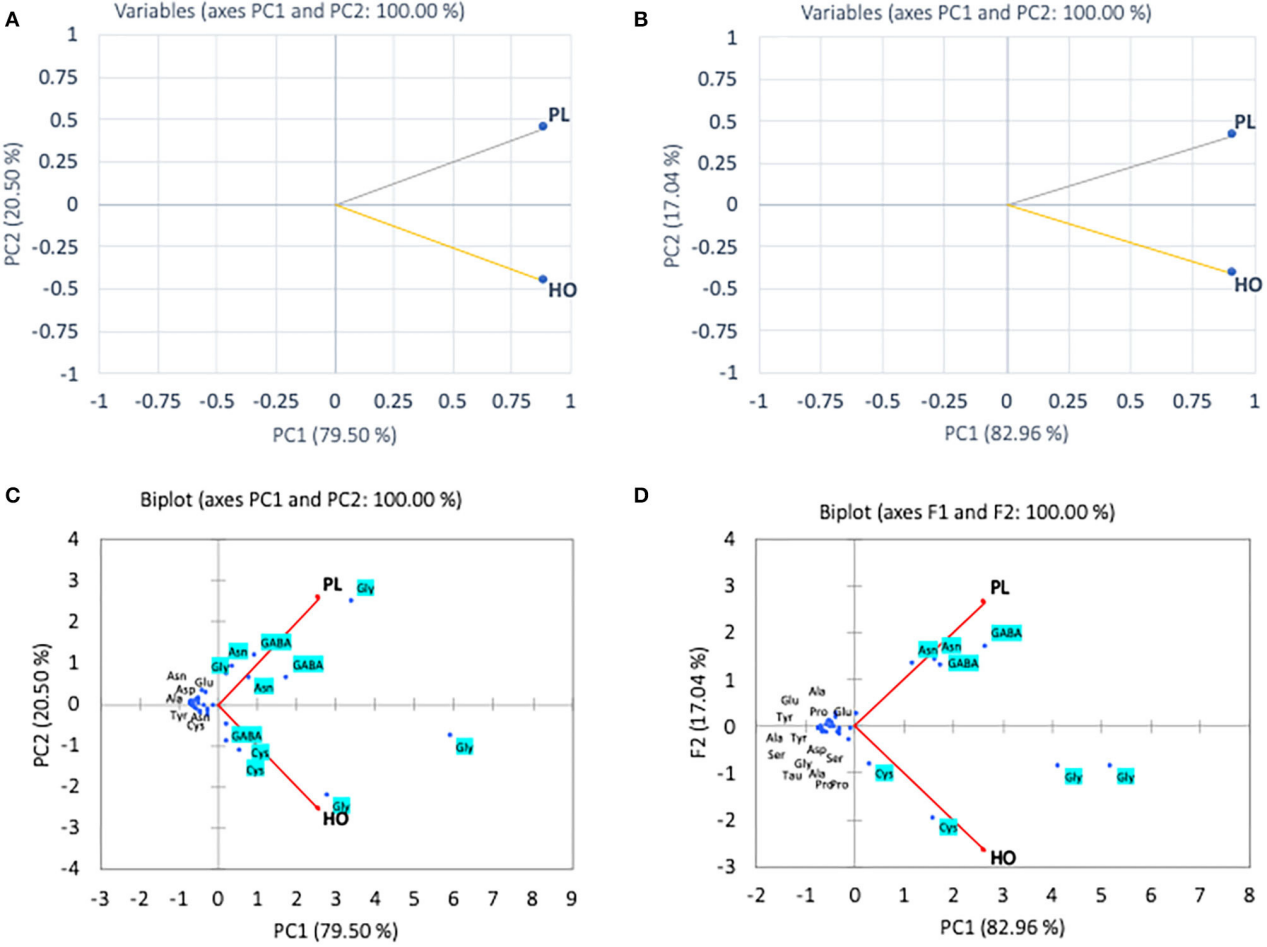
Principal component analyses of non-essential amino acid compositions of CL1–2 **(A)** and CL4–5 **(B)** and biplot cluster analysis with types of non-essential amino acids of CL1–2 **(C)** and CL3–4 **(D)** of pollen (PL) and honey (HO) of *A. andreniformis*.

In the pollen, cluster analysis of essential amino acid compositions of the colonies (Figure 2A) suggests that the compositions of CL3 was distinct from the others by the levels of Leu, Lys, Trp, and Val, whereas for non-essential amino acid compositions, the PCA clustered (i) CL3, (ii) CL4, CL5, and (iii) CL1, CL2 classes. It is apparent that within the second class, CL4 and CL5 related with the content of asparagine and GABA and were linked to the third class with Gly. The finding is in agreement with types of the pollen structures as indicated in Table 1, in which CL3 is found to have less variability of pollen sources from the others. To avoid drawing conclusion based on uncertain samples of CL3 was excluded for further analysis. The relationship between amino acid compositions of both honey and pollen of *A. andreniformis* are shown in Figures 3, 4. There was clear separation of amino acid compositions in these samples. Between the pollen and honey, the essential amino acids viz., Met, Phe, Leu, and Thr contributed some linkages in these two honeybee products (Figure 3B). For non-essential amino acids, the samples from each colony were PCA analyzed separately according to classes of amino acids profile from the pollen analyzed previously (Figures 2B,D). From the analyses, the results in Figures 4C,D revealed that even though there was discrete separation, the pollen amino acid profiles within sample classes (CL1, 2 and CL3, 4), asparagine, Cys, GABA, and Gly principally played some contributions to the non-essential amino acid linkage between the pollen and honey of *A. andreniformis*.

Amino acid profiles have been used to elucidate the origins of honey of different types (8, 10, 43), and pollen could largely contribute to the patterns of amino acids as honeybee collected nectar and pollen from the same botanical resources (3, 6). Pollen and honey are known as major sources of nutrient for bees. Worker bees consumed large amounts of pollen in order to fuel the growth and secretory activity of their hypopharyngeal glands (HGs), whereas foragers digest large amounts of nectar and honey to provide the energy for their flights (55). Pollen was collected, stored, and mixed with secretions from worker bees and then stored in the comb as beebread; the nutritional profile of the pollen is much higher by manner of storage (56). de Groot (40) and Taha et al. (12) illustrated ten amino acids that are essential for growth of the honeybee. Among those, our study claimed that from these particular botanical resources, Met, Phe, Leu, and Thr were abundant both in honey (nectar) and pollen for *A. andreniformis*.

**Figure 5:**
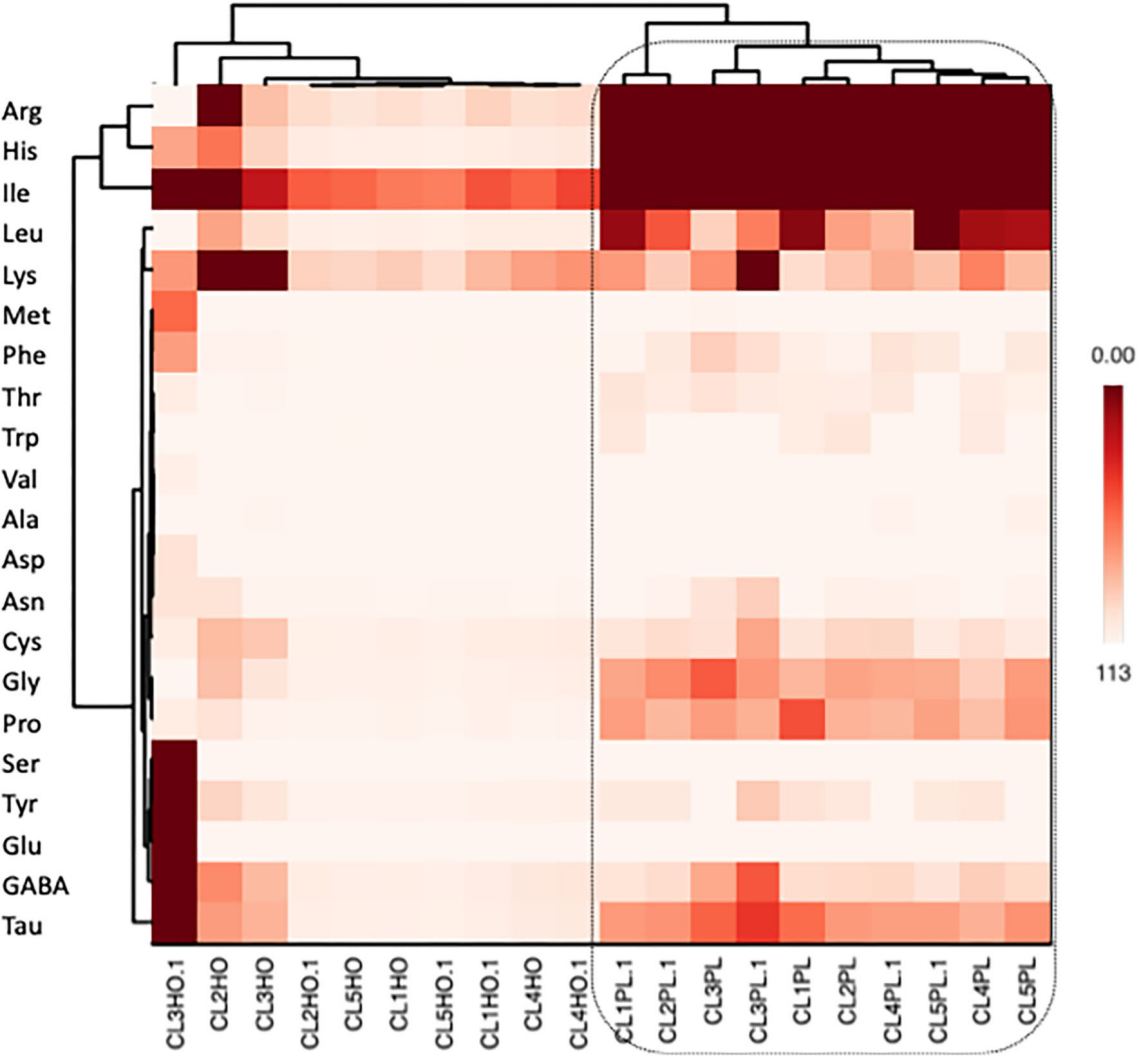
Amino acid profiling (heat mapping) of pollen and honey collected from different colonies of *A. andreniformis*.

Heat map was also performed to elucidate amino acid profiling of honey and honeybee pollen as well as their relationships (Figure 5). It is apparent that honey and honeybee pollen amino acids were distinct from one another.

### Proximate Composition

The results of proximate composition analyzed for both pollen and honey are displayed in Table 4. The pollens of nectar producing plants have an important role in determining the spectral percentage of pollen. The pollen concentration in a particular honey could serve a valuable tool in confirmation of its unifloral authenticity. A significant difference was found in moisture and carbohydrate content among the parameters of proximate analysis.

**Table 4:** Nutrition data of pollen and honeybee from *A. andreniformis*.

**Nutrition values**	**Pollen**	**Honey**	***p*-value**
Moisture (%)	**17.52** **±** **1.14**	**23.81** **±** **2.05**	**0.005**
Protein (%)	**13.50** **±** **1.11**	**2.11** **±** **0.45**	**0**
Ash (%)	0.57 ± 0.05	0.59 ± 0.05	0.722
Fiber (%)	**5.25** **±** **0.35**	**3.23** **±** **0.22**	**0.001**
Fat (%)	5.03 ± 0.02	Nil	—
Carbohydrate (%)	**57.86** **±** **2.63**	**70.27** **±** **2.70**	**0.05**
Energy (kcal/100 g)	**330.67** **±** **6.26**	**289.49** **±** **9.15**	**0.03**
Digestibility	0.85 ± 0.085	0.805 ± 0.18	0.81
EAAI	0.045	0.033	—
% AS	30.82	6.45	—

*Values are mean of the triplicates ± standard error. Bold indicates significant difference at 95% confidence*.

The moisture content varied from 21.52 (1) to 25.48% (3) in honey and 16.38 to 18.66% in pollen. The moisture content of the analyzed honey samples was found to exceed the limit as prescribed by Codex Alimentarius Commission that is <20%. Moisture content alone in honey and pollen is considered an important constituent that serves as a standard for its quality assessment in international trade (57). Moisture content has an important role in determining the quality of honey as it determines its shelf life and processing properties. Honey having more moisture is likely to be vulnerable to yeast spoilage (58). The low moisture content found in the pollen samples serves as a valid quality characteristic that protects it from spoilage caused by yeast fermentation. The moisture content of honey and pollen has been found to be determined by climatic conditions, geographical region, maturity period, plant sources, and temperature of storage area, harvesting time, and processing conditions (59). The crude fiber content ranged from 3.03 to 3.46% in honey and 5.16 to 5.85% in pollen. The presence of higher proportion of fiber in pollen could be attributed to its regulatory effect on intestinal functions as evidenced by clinical trials. The fiber content in pollen has been found to relieve the stomach problems by strengthening its linings and muscles, thereby preventing indigestion, colitis, constipation, and ulcers.

The ash content in honey varied from 0.56 to 0.65%, and in case of pollen, its content ranged from 0.52 to 0.63%. The lowest percentage of ash content indicates that pollen contains the lowest amount of minerals as ash content is related with the mineral content. The ash contents of the analyzed samples except honey of colony 3 and colony 1 pollen were observed to fall within the limits (<0.6%) prescribed by international norms (60). Thus, the CL3 honey and CL1 pollen could be regarded as Honeydew honey or a mixture of honeydew honey with blossom honey, whereas the other honey samples fall within the category of nectar honey as per standards of Codex Alimentarius Commission. The results of ash content in the present honey samples are in accordance with the values of honey samples from North-East Nigeria as reported by Buba et al. (61). The ash content (mineral content) in honey has been found by several researchers to be dependent mainly on its botanical origin and thus is considered an important constituent in determining the floral origin of honeys (62).

The protein content in honey was found to vary from 1.73 to 2.61% with honey from colony 3 showing the maximum protein content and honey of colony 1 the least. The protein content in the honey samples used in research was found to be higher than those analyzed by Buba et al. (61) from North-East Nigeria and in conformity with the honey samples analyzed by Agunbiade et al. (63). The higher amount of protein in pollen (12.3–14.5%) serves as the main protein and amino acid source for bees to enhance their gland development (64). The proteins in honey consist of two major groups including carbohydrate metabolism enzymes and royal jelly characteristic proteins (65). The proteins mainly present as enzymes in honey are responsible for the transformation of its chemical composition; in particular, the sugar spectrum. The number of amino acids in various honeys has been found to be 18, of which Pro predominates in almost all types of honeys.

Proteins in honey are mainly secreted from salivary and HGs of bees rather than from nectar (66). The diastases hydrolyze starch into maltose and is used an indicator for determining the freshness of honey. The Codex Alimentarius and the European honey directive have assessed eight diastase units for quality measurement in honey. Glucose oxidase and catalase present in honey possess antimicrobial effect as it regulates the production of hydrogen peroxide that acts as antibacterial agent. The proteins in honey possess antimicrobial activity and function as potential markers in determination of the geographical and botanical origin of honey (67). The significant differences observed between the protein contents of analyzed honey samples may be attributed to their different plant sources and bee species foraging on the same floral source (68).

The fat content analysis of the pollens used in this research were not found to differ significantly, with values ranging from 5.01 to 5.05%. Honeybees feed on pollen and plant nectar to produce honey. Pollen is an essential source of proteins, lipids, vitamins, and minerals, whereas as nectar is the main source of carbohydrates (69). The quality of the pollen diet reflects the nutrients available to honeybees and affects the physiological metabolism. It is not surprising that the honey analyzed in this study was observed to have zero fat content. The pollens with relatively higher fat content are considered to be lower in proteins and are useful in the attraction of foraging honeybees (70). Researchers have found that honey contains little to no fat content that correlates with the findings of the our study (71, 72). The carbohydrate content in the honey samples investigated in the present research was found to differ significantly from each other, with honey of colony 1 depicting the maximum content (73.16%), whereas the lowest carbohydrate content was found in honey of colony 3 having the value of 67.80%. Carbohydrates comprise the major portion of honey (about 82%), of which 38.2% is monosaccharide fructose and 31% glucose. The fructose and glucose are produced by hydrolysis of disaccharide sucrose, and the relative amount of these two monosaccharide's acts as an important criterion for the classification of unifloral honeys (73). The other carbohydrates comprise 25 different sugars including disaccharides, maltose, and sucrose (74). The result of the carbohydrate content obtained was found to be in accordance with the work of several researchers (63, 75).

The energy value of the honey samples ranged from 281.64 to 299.54 Kcal, and that of pollen samples varied from 324.49 to 337.0 Kcal. The energy in honey has been primarily attributed to its higher sugar content that are easily digestible (76). The enzymes in honey convert polysaccharides into monosaccharides and disaccharides that serve as essential energy sources. The present research showed the positive effect on *in vitro* digestibility of both pollen and honey samples. The digestibility percentage of pollen ranged from 0.841 to 0.864, whereas the honey samples were found to have digestibility percentage ranging from 0.620 to 0.887. The *in vitro* method is the precise and reliable method that depicts a physiologically relevant measure of digestibility of proteins. It plays an important role in understanding the capacity of the release of amino acids and peptides upon digestion of proteins and thus is one the quality indicators of proteins (77).

### Nutritional Profile of Pollen and Honey Proteins

The content of non-essential amino acids was observed to dominate the overall amino acid profile in all the analyzed honey and pollen samples of each colony. The pollen grains of all the colonies showed higher concentration of non-essential amino acids, whereas total essential amino acids were found to be highest in honey samples as elucidated in Table 2. Total essential amino acids were found to be highest in honey of colony 3, depicting value of 42.28, and the lowest value was shown by pollen of CL1. Honeybees cannot synthesis 10 amino acids that are essential for them and must be obtained from other food sources (57). The sole source of amino acids in diet of honeybees is the pollen, and not all these amino acids are provided by single types of pollen. Bees ingest the excess amount of pollen in order to recover the required amount of essential amino acids if the pollen is lacking the balanced amino acids (78). The lowest content (g/100 g) of essential amino acids in the pollens of CL1, 2, and 5 accounted for lowest total essential amino acids in honey of these colonies.

The pollen of CL3 was found to possess essential amino acid higher than the honeybee's requirements except Leu and Val, as depicted in Table 2. This leads to the higher concentration of the essential amino acids in honey extracted from CL3. The nutritional needs for honeybees depend on the composition of essential amino acids in pollen relative to requirements of honeybees as reported earlier by de Groot (40). Compared to the minimum requirements for adult humans, the essential amino acid composition of honey in CL3 was found to be superior in all the analyzed honey samples. From the calculated values of total essential amino acids, the higher percentage was shown by honey (42.28%) and pollen (4.48%) of colony 3, followed by CL4, 1, 2, and the least by CL5. The total non-essential amino acid content in the analyzed pollen samples was found higher than their corresponding honey samples, with pollen of CL1 showing higher percentage (97.82%) and that of CL3 the lowest (95.52%). The higher values of total acidic amino acids in honey of CL1 (6.04%) and CL2 (4.81) accounted for its extremely low pH and higher shelf life due to inhibiting growth of bacteria and other spoilage microorganisms (79). The branched-chain amino acids (BCCA) in all the analyzed honey samples was found to be the lowest (>1) except honey from CL5 (11.33) and CL3 (7.89), which indicates that the consumption of these honey samples (1, 2, and 4 colony) does not lead to the risk of development of type 2 diabetes and is safe for patients suffering from diabetic complications. Higher BCAA content has been reported to cause insulin resistance in humans, which results in development of diabetic complications (80).

The EAAI among the analyzed pollen and honey samples was found to be the highest in CL3 (27.989 and 61.44), which depicted it was having high-quality proteins with respect to essential amino acids. The EAAI gives a measure of protein quality that determines the content all essential amino acids with respect to a reference protein and human requirement as given by FAO/WHO standards (32). As each amino acid is essential for building the stable structure of protein, the rating of protein for assessment of nutritional quality must account for the whole contribution of essential amino acids. Thus, EAAI is a tool for representing the entire spectrum of essential amino acids with respect to nutritional standards (29).

BV indicates the proportion of absorbed protein that becomes incorporated into the proteins of the body cells was found highest in pollen and proteins of CL2 and CL3, respectively. The lowest BVs in honey samples from CL1, 4, and 5 indicated their capacity to be utilizable with the least fraction of the test proteins in cells of the organism. The lowest BV in these honey samples could be attributed to the missing some essential amino acids. The PER gives the estimation of comparing the food values of different proteins and was calculated on the basis of interaction between Leu–Pro and Leu–Tyr (PER-1 and PER-2). PER values are <1.5 in low-quality proteins and >2 in high-quality proteins as reported earlier by Anand et al. (81). The PER values were found the highest in honey of CL3 (>2.0), which could be due to the presence of higher content of essential amino acids.

### Amino Acid Scoring

Amino acid scoring is an indicator for measuring the efficiency of proteins and amino acids required for different population groups. It is based on the fact that protein synthesis by body cells is not done unless the required amino acids are taken by diet. Thus, amino acids score depicts the protein quality with respect to the proportion of essential amino acids present in a food material and that amino acids present in low quantity are considered as limiting amino acid (82). Ile was limiting amino acid in all the analyzed honey samples except honey from CL5 (Table 5). Lys was the limiting amino acid in all the pollen and honey samples, however the honey from CL3 was found to possess excessive concentration of Lys relative to FAO/WHO standard (32). Based on the essential amino acids scores for infants and adults, Leu, Thr, Met + Cys, and Phe + Tyr were the most prevalent essential amino acid in the pollen and honey of all colonies. The lowest values for chemical score represent the lesser quantity of amino acids as compared to the required amount.

**Table 5:** Amino acid score for infants/pre-school and adults.

	**FAO/WHO (32)**	**Colony 1**		**Colony 2**		**Colony 3**		**Colony 4**		**Colony 5**	
		**Pollen**	**Honey**	**Pollen**	**Honey**	**Pollen**	**Honey**	**Pollen**	**Honey**	**Pollen**	**Honey**
**Amino acid score (for infants/ pre-school 1–2 years)**											
Isoleucine	3.1	21.93	—	14.613	—	—	—	10.967	—	—	0.484
Leucine	6.3	14.76	3.87	11.81	14.92	16.190	125.23	7.301	5.39	6.03	2.698
Lysine	5.2	—	—	—	—	—	186.73	—	—	—	—
Methionine + cystine	2.6	1.07	89.69	96.81	304.23	429.61	343.08	161.54	139.61	122.31	58.54
Phenylalanine + tyrosine	4.6	20.87	10.913		30.652	47.17	33.26	31.74	11.95	14.565	5.859
Threonine	2.7	43.70	17.48	52.59	85.18	163.33	266.30	62.96	31.85	47.41	14.07
Valine	4.2	—	—	—	—	1.190	61.90	—	—	—	—
**Amino acid score (for adults)**											
Isoleucine	3.0	22.67	—	15.1	—	—	—	11.33	—	—	0.5
Leucine	5.9	15.76	4.135	12.61	15.93	17.28	133.73	7.797	5.763	6.441	2.881
Lysine	4.5	—	—	—	—	—	215.77	—	—	—	—
Methionine	1.6	17.5	3.25	28.56	7.5	98.75	115	33.12	1.875	46.25	0.75
Phenylalanine + tyrosine	3.8	25.26	13.21	39.23	37.10	57.10	35.26	38.42	14.47	17.63	7.105
Threonine	2.3	51.304	20.522	61.74	100	191.74	312.61	73.91	37.39	55.65	16.522
Valine	3.9	—	—	—	—	1.28	66.67	—	—	—	—
Cystine	0.6	416.66	380.00	343.33	1298.3	1598.3	1180.0	611.67	600.0	406.67	251.67
Histidine	1.5	—	—	—	—	—	37.333	—	—	—	—

## Conclusion

The structures of pollen gains identified from the sampling colonies showed monolete, inaperturate, trizonocolpate, dicolpate, monolate, and trilete forms. The pollens depicted greater non-essential amino acid contents than those of the honey, with Gly, GABA, and Asp being among the highest. The principal components were found to depict 60.87% and 19.80% of variance in case of essential amino acids. Between the pollen and honey, the essential amino acids viz., Met, Phe, Leu, Thr contributed some linkages in these two honeybee products. The high energy values in the analyzed honey samples have been primarily attributed to their higher carbohydrate contents. The present research validated that pollen morphology along with amino acid profile could be a valuable tool for discriminating pollen and honey from diverse botanical sources.

## Data Availability Statement

The data supporting the findings of the article are not publicly available, but it can be provided by the corresponding author on reasonable request.

## Author Contributions

SS designed the concept of this study. SS, BC, and MW drafted methodology. SS, BC, MW, TS, and PS conducted formal analyses and investigation. SS and FB wrote original draft of the manuscript. Writing-Review and Editing were completed by SS, FB, BC, MB, SS, and MB were project supervisors. Funding Acquisition was by SS. All authors contributed to the article and approved the submitted version.

## Conflict of Interest

The authors declare that the research was conducted in the absence of any commercial or financial relationships that could be construed as a potential conflict of interest.

## Abbreviations

AS: Amino acid score
EAAI: Essential Amino Acid Index
Ile: Isoleucine
Leu: Leucine
Phe: Phenylalanine
Thr: Threonine
Met: Methionine
Leu: Leucine
Trp: Tryptophan
Val: Valine
Asn: Asparagine
Gly: Glycine
Glu: Glutamic acid
GABA: Gamma-Aminobutyric acid
Cys: Cysteine
Pro: Proline
Ser: Serine
Asp: Aspartic acid
CL: Colonies.
